# In Patients with Early Peripheral Psoriatic Arthritis Baseline C-Reactive Protein, Pain and Ultrasound-Detected Synovitis Predict Subsequent Treatment with ts/bDMARDs. A Retrospective Analysis

**DOI:** 10.3390/jcm10132834

**Published:** 2021-06-27

**Authors:** Garifallia Sakellariou, Silvana Quaglini, Serena Bugatti, Francesca Bobbio-Pallavicini, Vittorio Gabba, Carlomaurizio Montecucco

**Affiliations:** 1 Department of Internal Medicine and Medical Therapy, University of Pavia, 27100 Pavia, Italy; serena.bugatti@unipv.it (S.B.); vittorio.gabba01@universitadipavia.it (V.G.); montecucco@smatteo.pv.it (C.M.); 2 Istituti Clinici Scientifici Maugeri, IRCCS, 27100 Pavia, Italy; 3 Department of Electrical, Computer and Biomedical Engineering, University of Pavia, 27100 Pavia, Italy; silvana.quaglini@unipv.it; 4 Division of Rheumatology, IRCCS Policlinico San Matteo Foundation, 27100 Pavia, Italy; f.bobbiopallavicini@smatteo.pv.it

**Keywords:** psoriatic arthritis, early arthritis, disease-modifying anti-rheumatic drugs, predictors

## Abstract

With the availability of effective treatment with targeted synthetic and biologic disease-modifying anti-rheumatic drugs (ts/bDMARDs) for psoriatic arthritis (PsA), it is crucial to identify predictors of access to this treatment since disease onset. We retrospectively enrolled patients with peripheral PsA, assessed in an early arthritis clinic from 2005 to 2020. The main baseline demographic, clinical and ultrasonographic (assessment of bilateral wrist and metacarpophalangeal joints) features were evaluated through descriptive statistics and tested as predictors by univariate and multivariate Cox models. The outcome of interest was the indication for ts/bDMARDs within 2 years from diagnosis. We included 238 patients with PsA, with a mean (sd) age of 51.04 (13.98) years; 90 (37.8%) were male, and the median (IQR) symptom duration was 6.12 (3.29–12.25) months. In univariate analyses, C-reactive protein (RR, 95% CI 1.204 (1.065,1.362)), Visual Analogue Scale (VAS) pain (1.027 (1.005,1.048)), the number of tender joints on 28 joints (1.087 (1.025, 1.153)), and a synovial power Doppler (PD) score > 1 (3.63 (1.307, 10.08)) emerged as significant predictors. C-reactive protein, VAS pain and PD confirmed their predictive value also in multivariate models. These results provide preliminary evidence on the features that might characterize patients with early peripheral PsA requiring more intensive monitoring and treatment escalation.

## 1. Introduction

Psoriatic arthritis (PsA) is a complex chronic inflammatory disease, in which a variety of clinical manifestations is present in combinations peculiar to each patient [1]. Besides heterogeneous patterns of joint involvement and typical extra-articular manifestations, patients with PsA experience an increased burden of comorbidities, cardiovascular events and mortality [2]. All of these aspects make PsA a complex and difficult-to-treat condition [3], in which the achievement of a satisfactory disease control still remains an uncovered need for many patients [4].

It is a matter of debate whether strategies of early diagnosis and management in PsA could have the same relevant impact as in rheumatoid arthritis (RA). In fact, despite evidence supporting the positive effect of establishing a diagnosis in the early phases of the disease, knowledge of early PsA is far less developed compared to early RA, and a clear definition of this condition has not yet been developed. So far, longer disease duration at presentation has been related to increased risk of radiographic progression, poorer physical function, and worse response to treatment [5,6,7,8]. However, there is still a limited number of observational studies with a specific focus on early PsA [9], and so far organizational models based on early diagnosis and intensive targeted treatment, widely available in the field of RA [10], have not been implemented for this disease. This might be one of the reasons for a still very considerable diagnostic delay in PsA [11]. Despite the presence of many gaps in the full understanding of the optimal management of early PsA, the Tight Control of psoriatic arthritis (TICOPA) study demonstrated a positive effect of a treat-to-target strategy with step-up disease modifying anti rheumatic drugs (DMARDs) applied in early phases of the disease [12]. Although the study reached a positive result, the magnitude of the effect was much smaller compared to similar studies in early RA, and very recently the results of the long-term follow-up of patients initially taking part to the trial failed to demonstrate a significant benefit at 5 years [13].

Besides a greater attention to the early identification of PsA, in the last few years there has also been a significant expansion of the number of licensed drugs for this condition, with an increasing number of approved targeted synthetic and biologic DMARDs (ts/bDMARDs), that are mainly indicated as a second-line treatment [14,15]. With the availability of effective treatments, the recognition of factors that could predict failure of first line therapy in patients with a recent diagnosis of PsA would be of great value in improving the management of this condition. In spite of the relevance of this aspect, however, only two studies have tried to identify predictors of access to ts/bDMARDs in patients newly diagnosed with PsA, showing an association between higher baseline C-reactive protein (CRP) and disease activity with the subsequent indication of second line treatment [16,17].

On the one hand, therefore, there is need for further evidence on early PsA to gain additional information on the impact of timely diagnosis and intensive targeted treatment. On the other hand, the identification of baseline predictors for failure of conventional approaches and indication to ts/bDMARDs would be of help in detecting patients with a more severe prognosis from the onset. The purpose of this study is therefore to describe and characterize a population of early peripheral PsA in the context of an Early Arthritis Clinic. In addition, we tried to identify baseline predictors of treatment with ts/bDMARDs within 2 years from diagnosis.

## 2. Materials and Methods

This study is based on the Early Arthritis Cohort of the IRCCS Policlinico San Matteo Foundation of Pavia, a prospective observational study including incident cases of inflammatory arthritis in the Pavia area, in Northern Italy. The Early Arthritis Clinic (EAC) was instituted in 2005, with a focus on RA, to assess patients with symptoms consistent with new-onset inflammatory arthritis of less than 12 months in duration [18]. Referral criteria were morning stiffness > 30 min, swelling of three or more joints and positive squeeze test of metacarpophalangeal or metatarsophalangeal joints [19]. At referral, patients underwent clinical assessment with joint counts on 28/44/68 joints for swelling and tenderness. The first symptom at onset (which might not correspond with the prevalent complaint at the time of the first assessment) was reported. Clinmetric variables (visual analogue scale (VAS) for general health (GH), pain, patient global assessment (PGA), fatigue, evaluator global assessment (EGA), the Italian version of the Health Assessment Questionnaire (HAQ)) were recorded [20]. Blood samples to measure erythrocyte sedimentation rate (ESR, range of normality < 20 mm/h) and CRP (range of normality < 0.5 mg/dl) were collected, as well as for the determination of anti-citrullinated protein autoantibodies (ACPA, range of normality < 10 U/mL) and rheumatoid factor (RF, range of normality < 20 U/mL). Conventional radiographs of the hands and feet were available at baseline in order to detect the presence of bone erosions. For the purpose of the present study, the EAC dataset was reviewed to retrieve all patients with an initial diagnosis of PsA, according to the Classification of Psoriatic Arthritis (CASPAR) criteria [21]. Disease activity was assessed through the Disease Activity Score on 28 joints (DAS) DAS28, and we also performed an exploratory analysis presenting the Disease Activity in Psoriatic Arthritis (DAPSA), although the PGA question in our cohort was that developed for RA. Patients with PsA were treated according to the current recommendations, and treatment was prescribed according to the prevalent manifestations. In particular, non-steroidal anti-inflammatory drugs (NSAIDs) were the first line treatment in patients with spondylo-arthritis, enthesitis, dactylitis and peripheral arthritis involving less than five joints. Local corticosteroid injections could be proposed for patients with mono-arthritis, dactylitis or enthesitis. In case of polyarthritis, patients were initially treated with DMARDs (including methotrexate, sulfasalazine, cyclosporine, leflunomide, and hydroxychloroquine). Low-dose glucocorticoids could be prescribed based on clinician’s decision. Patients were seen every two months in the first semester and every three afterwards, and treatment was adjusted according to clinician’s opinion in case of active disease. In patients reaching the maximal dosage of the different first-line treatment, in accordance with local regulations for the prescription of b/tsDMARDs and the available recommendations, depending on the main clinical manifestations, bDMARDs (including TNFα inhibitors, IL-17 inhibitors and IL-23 inhibitors) or tsDMARDs (apremilast) could be prescribed [15].

For this study, patients presenting before the end of 2020 were included. The study was conducted according to the Helsinki Declaration, and was approved by the Ethics Committee of the IRCCS Policlinico San Matteo Foundation. All patients signed a written informed consent form.

### 2.1. Ultrasonographic Evaluation

At the first evaluation, patients underwent ultrasound of bilateral wrists and metacarpophalangeal (1–5) joints, with high-level ultrasound equipment (GE Logiq9 from 2005 to 2010 (GE healthcare, Milwakee, US), ESAOTE MyLab70 Gold from 2010 (ESAOTE, Genoa, Italy)), with linear multi-frequency transducers, operating at 15–18 MHz to assess hand joints [18]. The joints were scanned on the dorsal side in longitudinal and transverse planes to detect synovitis and effusion, defined according to the Outcome Measures in Rheumatology (OMERACT) definition [22]. A large amount of gel was used to avoid pressure. The presence of power Doppler (PD) was confirmed in two perpendicular planes; the color box was set to include the joint and a variable area of surrounding tissues, including bony margins and the skin; pulse repetition frequency was set at 500–750 Hz, Doppler frequency was set high (7.5–14.3 MHz) and color gain was set just below the level causing random noise artifacts, to maximize sensitivity. Grey-scale (GS) (including both synovial hypertrophy and effusion) and PD were scored on a 0–3 scale for each joint, with overall scores (0–36) from the sum of single sites [23,24]. A single operator, a rheumatologist experienced in musculoskeletal ultrasound blinded to clinical findings, performed all assessments. GS and PD scores were treated both as continuous variables, and dichotomized with higher cut-offs, considering that mild abnormalities can often be found in healthy subjects [25]; in particular we selected a cut-off of PD > 1, based on previous studies [26] and of GS > 5, based on the distribution in our population, to select the most severe quartile.

### 2.2. Statistical Analysis

Descriptive statistics were applied to demographic and clinical data. For continuous data, mean and standard deviation (SD) or median and interquartile (IQR) range were used, based on parametric or nonparametric distribution of the variable, respectively. Categorical data were described by frequencies.

The event of interest was the initiation of a ts/bDMARD. Patients were observed until the prescription of ts/bDMARDs, until loss at follow up, until the last available date for patients seen after 2018 or until the second year from diagnosis. This time point was defined to include patients who most likely access a second-line treatment as a consequence of the intensification strategy in a tight-control and treat-to-target setting. The impact of baseline variables on the outcome was assessed through Cox models, reporting the results as Risk Ratios (RR) and 95% confidence intervals (95% CI). Some of the continuous variables were dichotomized to identify values with a possible higher predictivity; in particular, VAS pain was also assessed with a cut-off of 65 (chosen to identify severe pain [27,28]) and CRP of 0.6 mg/dl. Analyses were conducted initially by univariate models, and statistically significant variables (or those clinically relevant) were afterwards included in multivariate models with a number of predictors consistent with the “rule of thumb”, for which one additional predictor can be included every 10 events. Survival until the initiation of ts/bDMARDs was also presented through Kaplan-Meier curves, compared by the log-rank test. Significance was set at 0.05.

## 3. Results

### 3.1. Descriptive Analyses

From the larger population of the EAC cohort, 238 patients with PsA were identified and included in the present analysis. The mean (SD) age was 51.04 (13.98) years, 90 (37.8%) patients were male, and median (IQR) symptom duration was 6.12 (3.29–12.25) months. 168 (78.5%) patients had current psoriasis or a history of psoriasis. The median values of CRP and ESR were within the range of normality, and the mean (SD) DAS28 was 3.74 (1.23), consistent with moderate disease activity. 155 (67.4%) patients were initially treated with csDMARDs, in particular 80 (34.8) received methotrexate and 58 (25.2%) sulfasalazine; 60 (25.3%) also received low-dose glucocorticoids. The detailed demographic and clinical features at baseline are reported in Table 1.

**Figure 1:**
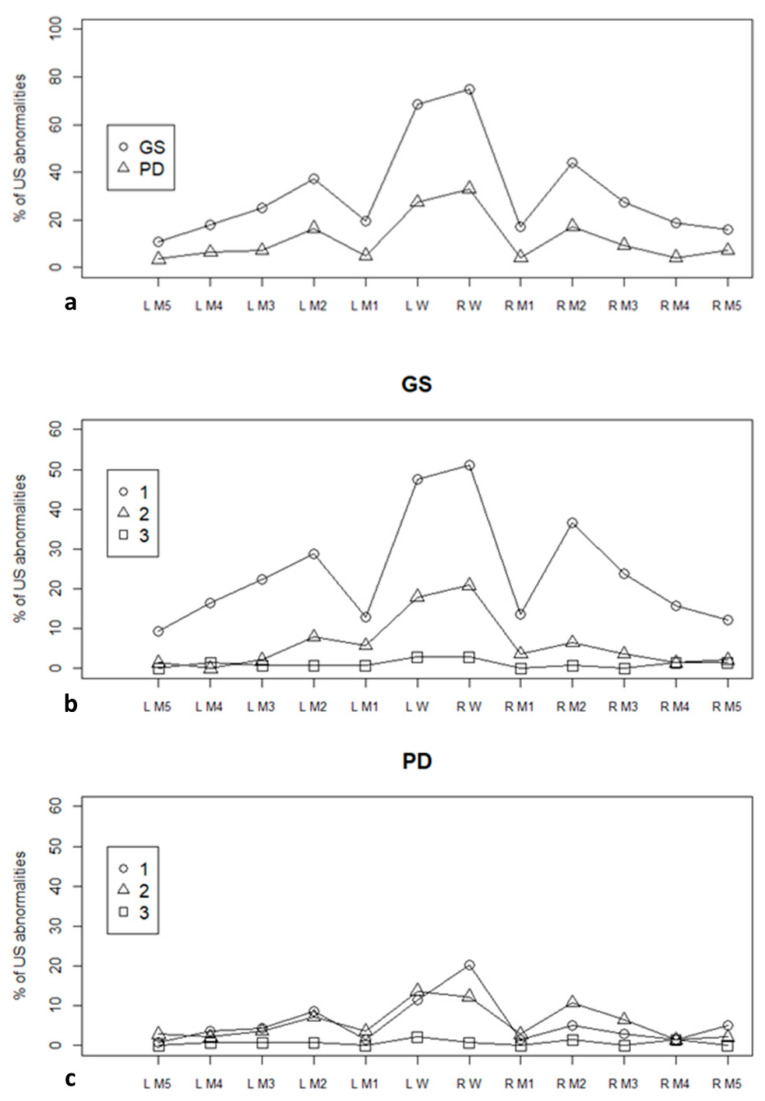
Prevalence of ultrasonographic abnormalities compared to the total number of scanned joints, according to the joint site and the type of lesion. (**a**) proportion of ultrasonographic abnormalities of any grade; (**b**) proportion of grey scale abnormalities, presented according to the grade of severity; (**c**) proportion of power Doppler abnormalities, presented according to the grade of severity. US: ultrasound; GS: grey scale; PD: power Doppler; L: left; R: right; M: metacarpophalangeal joint; W: wrist.

The median (IQR) GS score was 4 (2–6) in our population, while the median (IQR) PD score was 1 (0–3). The prevalence of ultrasound-detected abnormalities, overall (Figure 1a) and depending on the severity (Figure 1b,c), is presented in Figure 1.

### 3.2. Predictors of bDMARD or tsDMARD Treatment

**Figure 2:**
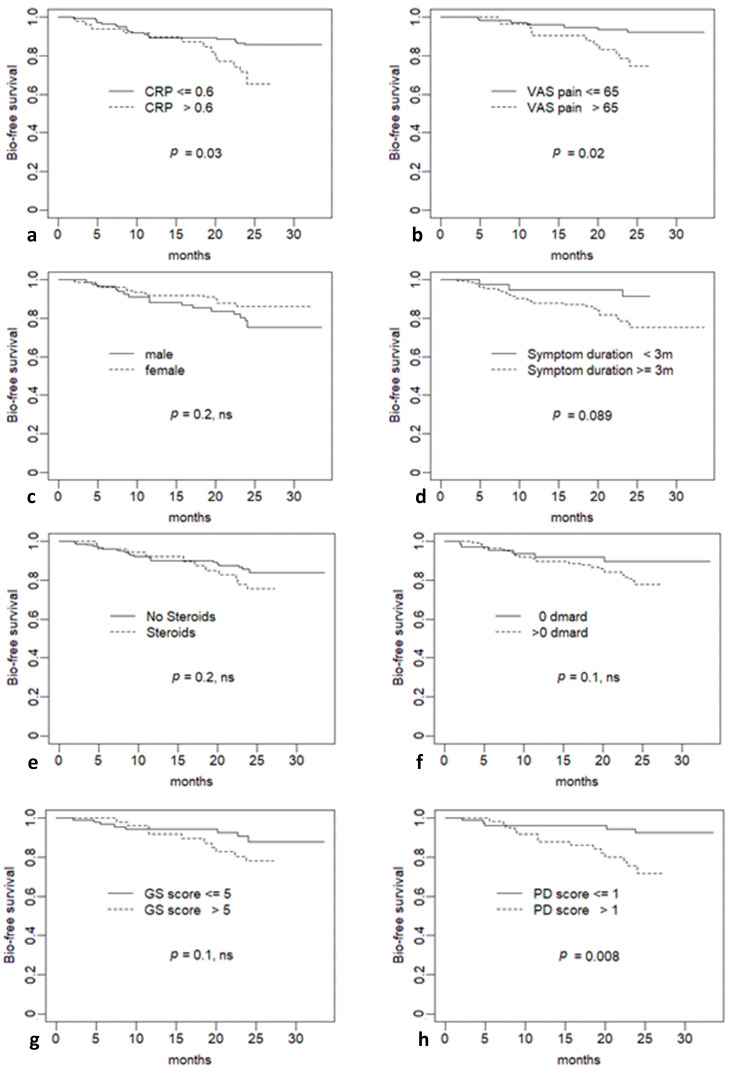
Survival curves (outcome: initiation of ts/bDMARDs) for baseline variables. (**a**) C reactive protein; (**b**) visual analogue scale for pain pain; (**c**) gender; (**d**) symptom duration; (**e**) treatment with glucocorticoids; (**f**) tratment with csDMARDs; (**g**) ultrasound grey scale score; (**h**) ultrasound power Doppler score. M: months; CRP: C reactive protein; VAS: visual analogue scale; DMARD: disease modifying anti rheumatic drug; GS: grey scale; PD: power Doppler.

Within a median (IQR) follow-up of 24.01 (9.74–24.03) months, 32 patients started treatment with bDMARDs or tsDMARDs. In univariate analyses, among baseline characteristics, age, gender, disease duration and personal history of psoriasis did not emerge as significant predictors of second-line treatment. In contrast, baseline values of CRP proved to be significant predictors, both when evaluated as continuous variable (RR, 95% CI 1.204 (1.065,1.362)) and as categorical variable, for values > 0.6 mg/dl (RR, 95% CI 2.137 (1.047,4.362)) (Figure 2a). Among clinimetric variables, VAS pain was the only one to result in a significant prediction, both as continuous variable (RR, 95% CI 1.027 (1.005,1.048)) and as a categorical variable, with a cut-off of 65 mm (RR, 95% CI 3.02 (1.17,7.798)) (Figure 2b). In line with this finding, the number of tender joints also predicted second line treatment. Among ultrasonographic variables, although PD score as continuous variable was not a significant predictor, a baseline PD score > 1 predicted indication to second line drugs (RR, 95% CI 3.63 (1.307,10.08)). Type of initial treatment, including treatment with csDMARDs, was not a significant predictor. This result was also consistent when excluding patients treated with hydroxychloroquine from the analysis on overall csDMARDs (RR, 95%CI 2.056 (0.889,4.757)). Table 2 reports in detail the results of the univariate analyses, while Figure 2, panels c–h).

Comparable results were obtained by comparing survival curves, as shown in Figure 2.

We tested a multivariate model including three variables. Due to the likely correlation between tender joint counts and VAS pain, and CRP and PD score (Spearman’s correlation coefficient 0.169, *p* 0.04), we included in the model VAS pain, PD score as categorical variable, and gender (not significant in univariate analyses, but known to be a predictor of relevant outcomes in previous studies). While VAS pain and PD score were still significant predictors after adjustment, female gender also resulted in a significant association with treatment (Table 3).

## 4. Discussion

Although in recent years there has been an increasing interest in the impact of a timely diagnosis and intensive targeted treatment in PsA, leading to relevant strategic studies such as the TICOPA [12], there is still a significant gap in our knowledge on how this disease should be handled at its initial stages. In particular, while it has been demonstrated that early diagnosis leads to more favorable outcomes, the effort to create dedicated tracks, as already largely implemented in RA, has so far been very limited. In this study, we provide one of the first descriptions of a population of peripheral PsA enrolled in the context of an early arthritis clinic. Although we collected data retrospectively from a cohort that was initially conceived to study early RA, the size of the sample that we retrieved is comparable to that of cohort studies specifically developed for early PsA [8], and the overall demographical and clinical baseline characteristics are in line with those previously reported, although there are a few, but very relevant, discrepancies. First, the duration of symptoms in our population is relevantly shorter than in previous studies, with a median symptom duration of about 6 months [8]. Although this finding might have been conditioned by the inclusion criterion in the EAC (a disease duration of less than 12 months), it suggests a potential impact of a dedicated track on an earlier diagnosis of peripheral PsA. Moreover, and this could be a consequence of the shorter symptom duration, the median values of acute phase reactants in our population are lower and still within the range of normality, suggesting a limited impact of systemic inflammation. The majority of our patients required initial treatment with csDMARDs, while only a minority received glucocorticoids. While the most represented subset of disease was peripheral oligo-arthritis, most of our patients had received NSAIDs before referral: the failure of this first line approach might account for the frequent prescription of csDMARDs. The remaining demographic and clinical features at baseline were comparable to those of the SwePsA cohort, despite the recruitment criteria being initially designed for RA [8].

In our population, 32 patients required treatment intensification, consistently with previous reports [16], with the indication for ts/bDMARDs. Among baseline demographic and clinical features, in line with previous studies, CRP, both as continuous variable and with the cut-off of 0.6 mg/dl, resulted in a significant prediction of second line treatment [17]. Moreover, joint pain seemed to condition failure of first line treatment, since both VAS pain, as a continuous variable and with the cut-off of 65 mm, and the number of tender joints were shown to be significant predictors of ts/bDMARD initiation. Our results are in line with the emerging relevance of pain management as an unmet need in PsA, with this component acting as a driver for treatment intensification [29].

Very interestingly, baseline ultrasound variables were able to identify patients with a more severe course. In fact, patients presenting with PD positive synovitis at the wrist and metacarpophalangeal joints were more subject to receiving ts/bDMARDs. We chose the cut-off of 1 for the score of PD synovitis because low-degree signals can be seen in healthy subjects and they are not indicative of active synovitis in arthritis. So far, a very limited number of studies has specifically investigated the prognostic value of ultrasound findings in PsA, and the lesion of greater interest for this purpose was enthesitis [30]. In our population, the ultrasound protocol was limited to the evaluation of synovitis in a small number of joints, which is more suitable in the setting of RA. In spite of this limitation, information deriving from ultrasound seemed to be of value in identifying patients with a poorer prognosis, although this finding will need confirmation in prospective studies [31].

While existing data related disease activity to the indication for ts/bDMARDs, our results did not confirm this finding, although baseline mean DAS28 was comparable to that of previously described populations [16]. Of note, the possibility to consider PsA-specific disease activity indexes in order to fully compare our work to previous studies was limited, although DAS28 has been validated in polyarticular PsA [32]. In fact, although we tested the predictive value of DAPSA in our cohort, this was an exploratory analysis due to the available version of the PGA. This was also due to the fact that DAPSA has been validated after the establishment of the EAC [33]. In addition, the follow-up was longer in existing studies. Moreover, while in the past symptom duration has been related to more favorable outcomes, in our analysis it did not significantly predict a more limited use of second line treatment [5]. The results of our study do not suggest the presence of a window of opportunity in peripheral PsA, in the specific context of a disease duration of less than 12 months, and this could be related to a slower progression towards chronicization, compared with RA.

The possibility of assessing independent predictors in our cohort was limited by the relatively low number of events, which did not allow us to construct solid multivariate models with more than three predictors. We included in multivariate models those variables resulting in a significant prediction in univariate analysis, excluding the number of tender joints, likely correlated with VAS pain, and CRP, which correlates to ultrasound-detected synovitis [33]. We used this approach to try and avoid the presence of collinearity in the models. We included gender, which had proved to be a relevant predictor in previous studies. From this model, while VAS pain and PD positive synovitis were confirmed to be significant predictors, gender also emerged as a candidate predictor. In particular, female gender seemed to play a protective effect against our outcome, and this is in contrast with previous reports, in which males tended to fare better for other outcomes [8]; previous studies using the same outcome, however, reported no influence of gender [16]. This finding will therefore need confirmation in subsequent studies.

Our study has several limitations, mostly due to the retrospective design and to the fact that the EAC was first conceived as an observational study on RA, and this reflects the fact that all measures are RA-specific. Inclusion criteria are meant to identify a different disease, therefore we mainly enrolled patients with PsA with peripheral joint involvement, with poor representation of patients with axial disease or entheso-arthritis. Measures useful to describe PsA (type of involvement, dactylitis, enthesitis, severity of skin and nail involvement) are not present in our dataset, as well as information on intra-articular glucocorticoid injections; and the measures of disease activity are those validated for RA. The ultrasound protocol that we adopted considers a limited number of joints and lesions, not assessing the value of enthesitis and tenosynovitis, and this might not always reliably reflect disease activity in PsA. A further limitation is the small number of events that did not allow the exploration in detail of all potential confounders, or the introduction of many variables in multivariate models in order to assess their potential independency. Due to these limitations, the significantly related variables can more reliably be seen as associated with the outcome, rather than as strong predictors.

In spite of these limitations, the relevance of this work is that it enriches the available information on early peripheral PsA, by adding data from a large population with a particularly short disease duration. Our cohort has the unique peculiarity, in this field, of presenting both clinical and ultrasound data at disease onset, and, to our knowledge, such information is currently not available in any other large observational study. Moreover, this study has the value of assessing the features of PsA patients evaluated in the context of an early arthritis clinic.

## 5. Conclusions

Our study provides one of the few available characterizations of early peripheral PsA, suggesting that organizational models, such as early arthritis clinics, might also have a positive impact on the management of PsA. Higher baseline pain, CRP and the presence of ultrasound-detected synovitis might help identify those patients for whom a more intensive monitoring and treatment escalation could be useful. These results could be confirmed by subsequent prospective research.

## Figures and Tables

**Table 1:** Baseline characteristics of the included population.

*n*	238
M/F (*n*, %)	90/148 (37.8)
Age (mean, sd)	51.04 (13.98)
First symptom (*n*, %)	
Inflammatory pain	108 (54)
Polyarticular	40 (20)
Oligoarticular	25 (12.5)
Monoarticular	25 (12.5)
Morning stiffness	1 (0.5)
Other	1 (0.5)
Symptom duration, months (median, IQR)	6.12 (3.29–12.25)
Psoriasis (*n*, %)	168 (78.50)
Previous treatment (*n*, %)	119 (52.19)
NSAIDs	82 (68.90)
Glucocorticoids	30 (25.2)
csDMARDs	7 (5.9)
Pattern of joint involvement at referral (*n*, %)	
Polyarticular	70 (31.5)
Oligoarticular	94 (42.3)
Monoarticular	41 (18.5)
No joint swelling	17 (7.7)
CRP, mg/dl (median, IQR)	0.23 (0.06–0.77)
ESR, mm/h (median, IQR)	11 (6–20.75)
Rheumatoid Factor (*n*, %)	14 (6.7)
ACPA (*n*, %)	2 (0.9)
ANA (*n*, %)	90 (39.8)
VAS PGA, mm (median, IQR)	50 (30–70)
VAS pain, mm (median, IQR)	50 (34.50–70.50)
VAS fatigue, mm (median, IQR)	60 (20–80)
VAS GH, mm (median, IQR)	60 (45–75.75)
VAS EGA, mm (median, IQR)	32.50 (20–45)
HAQ (median, IQR)	0.75 (0.37–1.12)
HAQ > 0.5 (*n*, %)	106 (61.6)
Duration of morning stiffness, minutes (median, IQR)	30 (10–60)
Tender joint count (median, IQR)	
28 joints	3 (1–7)
44 joints	5 (2–12)
68 joints	5 (2–11.50)
Swollen joint count (median, IQR)	
28 joints	2 (1–4)
44 joints	3 (1–6)
66 joints	2 (1–4)
RAI (median, IQR)	4 (2–7)
DAS28 (mean, sd)	3.74 (1.23)
DAS44 (mean, sd)	2.42 (0.86)
DAPSA (median, IQR)	21.45 (14.11–31.53)
csDMARDs (*n*, %)	155 (67.4)
Methotrexate	80 (34.8)
Cyclosporine	6 (2.6)
Sulfasalazine	58 (25.2)
Hydroxychloroquine	12 (5.2)
Glucocorticoids (*n*, %)	60 (25.3)
Glucocorticoids, dose (median, IQR)	5(0–5)
GS score (median, IQR)	4 (2–6)
GS score > 5 (*n*,%)	53 (34.4)
PD score (median, IQR)	1 (0–3)
PD score > 1 (*n*, %)	65 (41.7)
Follow-up, months (median, IQR)	24.01 (9.74–24.03)

*n*: number; M: male; F: female, IQR: interquartile range; sd: standard deviation; NSAIDs: non-steroidal anti-inflammatory drugs; csDMARDs: conventional synthetic disease modifying antirheumatic drugs; CRP: C reactive protein; ESR: erythro-sedimentation rate; ACPA: anti-cyclic citrullinated peptide antibodies; ANA: anti-nuclear antibodies; VAS: visual analogue scale; PGA: patient’s global assessment; GH: general health; EGA: evaluator’s global assessment; HAQ: Health Assessment Questionnaire; RAI: Ritchie Articular Index; DAS28: disease activity score on 28 joints, DAS44 disease activity score in 44 joints; GS: Grey Scale; PD: power Doppler.

**Table 2:** Results of the univariate Cox regression analyses.

	Univariate	
Predictors	Risk Ratio (95% CI)	*p*-Value
Female gender	0.607 (0.304,1.215)	0.159
Symptom duration ≥ 3 months	2.815 (0.852,9.305)	0.089
Age	0.979 (0.956,1.004)	0.102
Psoriasis	1.038 (0.449,2.401)	0.93
CRP	1.204 (1.065,1.362)	0.003
CRP > 0.6	2.137 (1.047,4.362)	0.037
ESR	0.984 (0.955,1.014)	0.297
DAS28	1.116 (0.768,1.621)	0.564
DAS44	1.249 (0.738,2.115)	0.407
DAPSA	1.029 (0.995,1.064)	0.090
VAS pain	1.027 (1.005,1.048)	0.014
VAS pain (>65 vs. ≤65)	3.02 (1.17,7.798)	0.022
VAS fatigue	1.014 (0.997,1.031)	0.095
VAS GH	0.995 (0.977,1.014)	0.63
VAS PGA	1.017 (0.998,1.037)	0.077
Tender joint count/28	1.087 (1.025,1.153)	0.005
Tender joint count/44	1.038 (0.998,1.079)	0.063
Tender joint count/68	1.06 (1.023,1.099)	0.001
Swollen joint count/28	1.047 (0.957,1.145)	0.317
Swollen joint count/44	0.996 (0.916,1.082)	0.992
Swollen joint count/66	1.045 (0.961,1.135)	0.305
RAI	1.058 (0.969,1.154)	0.209
HAQ	0.907 (0.383,2.152)	0.826
HAQ > 0.5	1.375 (0.484,3.906)	0.550
Bone erosions	1.863 (0.791,4.385)	0.154
GS score	1.081 (0.984,1.189)	0.106
GS score > 5	1.992 (0.785,5.05)	0.147
PD score	1.072 (0.981,1.171)	0.123
PD score > 1	3.63 (1.307,10.08)	0.013
Glucocorticoids	1.547 (0.746,3.208)	0.241
csDMARDs	1.812 (0.775,4.235)	0.17
Methotrexate	1.942 (0.971,3.886)	0.061
Hydroxychloroquine	0.475 (0.065,3.483)	0.464
Cyclosporine	3.231(0.767,13.61)	0.11
Sulfasalazine	0.741 (0.320,1.714)	0.483

CI: confidence interval; CRP: C reactive protein; ESR: erythro-sedimentation rate; VAS: visual analogue scale; PGA: patient’s global assessment; GH: general health; EGA: evaluator’s global assessment; HAQ: Health Assessment Questionnaire; RAI: Ritchie Articular Index; DAS28: disease activity score on 28 joints, DAS44 disease activity score in 44 joints; GS: Grey Scale; PD: power Doppler; csDMARDs: conventional synthetic disease modifying antirheumatic drugs.

**Table 3:** Results of the multivariate Cox regression analyses. CI: confidence interval; VAS: visual analogue scale; PD: power Doppler.

	Multivariate	
Predictors	Risk Ratio (95% CI)	*p*
VAS Pain	1.031 (1.006,1.056)	0.012
PD Score > 1	3.899 (1.067,14.252)	0.039
Female Gender	0.325 (0.108,0.977)	0.045

## Data Availability

The data presented in this study are available on reasonable request from the corresponding author.
